# Clinical and Oncological Outcomes after Uniportal Anatomical Segmentectomy for Stage IA Non-Small Cell Lung Cancer

**DOI:** 10.3390/medicina59061064

**Published:** 2023-06-01

**Authors:** Konstantinos Gioutsos, Yves J. Hayoz, Patrick Dorn

**Affiliations:** Department of Thoracic Surgery, Inselspital, University Hospital of Bern, 3010 Bern, Switzerland

**Keywords:** sublobar resection, uniportal, segmentectomy, stage IA NSCLC, lung cancer

## Abstract

*Background and Objectives*: The existing literature comparing sublobar and lobar resection in the treatment of stage IA lung cancer highlights the trend and overall need for further evaluation of minimally invasive, parenchymal-sparing techniques. The role of uniportal minimally invasive segmentectomy in the oncological therapy of early-stage non-small cell lung cancer (NSCLC) remains controversial. The aim of this study was to evaluate the clinical and midterm oncological outcomes of patients who underwent uniportal video-assisted anatomical segmentectomy for pathological stage IA lung cancer. *Materials and Methods*: We retrospectively analyzed all patients with pathological stage IA lung cancer (8th edition UICC) who underwent uniportal minimally invasive anatomical segmentectomy at our institution from January 2015 to December 2018. *Results*: 85 patients, 54 of whom were men, were included. The median length of hospital stay was 3 days (1.-3. IQR 3–5), whereas 30-day morbidity was 15.3% (13 patients), and the in-hospital mortality rate was 1.2% (1 patient). The 3-year overall survival rate was 87.9% for the total population. It was 90.5% in the IA1 group, 93.3% in the IA2 group, and 70.1% in the IA3 group, respectively. *Conclusions*: There were satisfactory short-term clinical outcomes with low 30-day morbidity and mortality and promising midterm oncological survival results following uniportal minimally invasive anatomical segmentectomy for pathological stage IA non-small cell lung cancer.

## 1. Introduction

It is well known that lobectomy is the standard of care for surgically treatable early-stage lung cancer [1]. However, the importance of sublobar pulmonary resections has steadily increased due to the aging population with increasing comorbidities. Especially for small (<2cm) peripheral lung tumors, anatomical segmental resections are increasingly considered equivalent to lobectomy regarding recurrence and oncologic outcome [2,3]. Patients considered high-risk for surgery due to impaired lung function or comorbidities and who would not otherwise tolerate a lobectomy are likely candidates for anatomical sublobar resections, potentially expanding therapeutic options for a patient population previously considered inoperable. The anticipated advantages of minimally invasive sublobar lung resections in otherwise inoperable patients are primarily two. One is the lung-sparing nature of the segmentectomy, with its preservation of more lung tissue; the other is the reduced mechanical trauma to the chest wall from the minimally invasive thoracoscopic technique, and even more so from the single-incision technique. The potential advantage of uniportal VATS is less postoperative pain [4]. However, comparisons with multiportal VATS are inconclusive. In terms of blood loss, duration of drainage, and length of hospital stay, uniportal VATS appears to be equivalent or perhaps superior to multiportal VATS [4,5].

According to the American College of Chest Physicians [6], pulmonary segmentectomy should be considered for peripheral lung tumors smaller than 2 cm and without lymph node involvement in patients not medically suitable for lobectomy. The use of sublobar resection as an alternative to lobectomy for early-stage non-small cell lung cancer (NSCLC) has been debated for many years; however, current guidelines for treating stage IA NSCLC still recommend lobectomy in patients who can tolerate it. This recommendation is based on a Lung Cancer Study Group (LCSG) study from 1995, which showed that limited resection in patients with peripheral T1 N0 NSCLC was associated with a higher mortality rate and a higher rate of locoregional recurrence [1]. However, it should be noted that non-anatomical wedge resections were also included.

Many recent studies have shown that segmentectomy is oncologically non-inferior to lobectomy for small solid lung tumors [7,8,9], and there are comparable oncological outcomes after segmentectomy in stage I NSCLC, particularly in patients with T1a NSCLC who are not eligible for lobectomy [10,11,12].

Developments in imaging technology and the widespread use of thin-slice computed tomography (CT) for screening purposes have simplified the diagnosis of early-stage lung cancer [13]. Such lesions may be overtreated with the standard method of lobectomy. The long-awaited randomized controlled trial from the Japan Clinical Oncology Group (JCOG0802/WJOG4607L) showed that segmentectomy was superior to lobectomy in terms of overall survival and recurrence-free survival by early-stage, node-negative lung cancer. This was the first randomized trial comparing segmentectomy with lobectomy for peripheral N0 lung cancer ≤2 cm in size. The authors concluded that segmentectomy should be the standard surgical procedure for these lesions [14]. These findings were also confirmed by the results of the multicenter, international, non-inferiority, phase 3 trial of Cancer and Leukemia Group B (CALGB140503), which enrolled 83 centers from the United States, Canada, and Australia [15].

In terms of clinical outcome, previous studies have shown that the complication rate after segmentectomies is comparable to that after lobectomies, except for a higher incidence of air leakage after segmentectomies [16,17]. Moreover, video-assisted thoracoscopic surgery (VATS) is a safe alternative to open surgery without compromising oncological efficacy. It is associated with fewer perioperative complications, less postoperative pain, faster recovery, and better quality of life than lobectomy by thoracotomy [18,19]. In recent years, single-portal (uniportal) VATS has gained acceptance in the treatment of early-stage NSCLC. Although initial studies suggest favorable outcomes for uniportal VATS in lung cancer treatment, long-term follow-up studies are still needed to verify the role and potential benefits of uniportal lung surgery [18,20,21]. In particular, the role of uniportal VATS segmentectomy in the oncological treatment of early-stage lung cancer remains controversial.

Currently, the indication for intended segmentectomy for lung cancer is limited to only peripheral T1 (≤2 cm) N0 lesions. Moreover, compromised segmentectomies are ‘accepted` for poor lung function, synchronous lung cancer, or severe comorbidity.

In 2015, uniportal VATS segmentectomies were introduced at our institution, gradually replacing traditional three-port VATS segmentectomies.

Our study aimed to retrospectively analyze all patients who underwent anatomical segmentectomy with the uniportal VATS technique for pathological stage IA NSCLC (8th edition) between 2015 and 2018 at our institution in terms of clinical and midterm oncological outcomes.

## 2. Materials and Methods

This study was conducted according to the guidelines of the Declaration of Helsinki (as revised in 2013) and approved by the Ethics Committee of the Canton of Bern, Switzerland (2022-00673). This article was written following the STROBE guidelines (Strengthening the Reporting of Observational Studies in Epidemiology).

### 2.1. Patients

This study was a single-center retrospective analysis of all patients who underwent uniportal thoracoscopically assisted pulmonary segmentectomy for pathological stage IA (stage IA1-3, 8th ed. UICC) (NSCLC) at our institution from 1 January 2015 to 31 December 2018. We analyzed the pre-, peri-, and postoperative data of all patients. We excluded from the analysis all patients who were upstaged due to either lymph node involvement or tumor size.

Operative data such as tumor size and stage, histological subtype, number of lymph node stations harvested, lymph nodes affected, and duration of surgery were documented.

We evaluated the duration of chest drainage and hospital stay, 30-day morbidity, and postoperative mortality.

Oncological follow-up after surgical therapy for early-stage cancer was performed at fixed intervals by chest CT scan. At our institution, CT scans are performed every six months for the first two years after surgery and then annually for at least five years. Regarding oncological follow-up, the data were considered until 30 April 2022. All these data were obtained from the medical information system of our hospital. All patients gave their written consent for further use of health-related data.

### 2.2. Endpoints

As primary endpoints, we analyzed 30-day morbidity and 3-year overall survival. Secondary endpoints were tumor-related deaths and tumor recurrence, locoregional or distant.

### 2.3. Indications

The indications for segmentectomy at our institution were as follows:

Intended segmentectomy was performed only in patients with peripheral tumors less than 2 cm in size without lymph node involvement.

Patients with poor lung function, synchronous lung cancer, and severe comorbidity that would make the risk of severe complications or even death perioperatively unacceptable underwent compromised resections.

The choice of surgical procedure was based on tumor size and preoperative staging from an oncological perspective. At a second level, the patient’s functional operability was assessed by lung function tests and preexisting comorbidities. This decision was discussed in an interdisciplinary manner at the thoracic tumor board and was verified again in a second discussion between board-certified thoracic surgeons.

In the case of lesions 2–3 cm located centrally in a lung segment, we would proceed with a segmentectomy of 2 or 3 segments, depending on what is technically appropriate.

### 2.4. Operative Technique

We used the standard uniportal VATS approach as previously described [22,23]. All operations were performed by one of the board-certified general thoracic surgeons at our institution. Patients were intubated (one-lung ventilation) and placed in the lateral decubitus position. A 3–4 cm incision was made in the 4th or 5th intercostal space (anterior or midaxillary line), and a ring wound protector was placed. A 5mm 30-degree scope and dedicated uniportal VATS instruments were used. The bronchovascular structures (segmental artery, bronchus, and vein) were dissected and transected using endoscopic staplers or vascular clips. The intersegmental plane was completed with staplers. All patients underwent systematic locoregional, hilar, and mediastinal lymph node dissection.

### 2.5. Statistical Analysis

Quantitative variables were expressed as means and standard deviation (SD). Qualitative variables were expressed as absolute (N) and relative frequencies (%). Overall survival was calculated using the Kaplan–Meier method, and the log-rank test was used to assess survival differences between groups. All statistical analyses were performed using Stata version 16. The aforementioned statistical tests were performed at a significance level of 0.05.

## 3. Results

### 3.1. Demographics

During the study period, 222 uniportal VATS segmentectomies were performed. From this cohort, 85 of the patients that underwent surgery for pathological stage IA NSCLC. 54 (63.5%) were men and had a mean age of 66.4 years.

A total of 69 (81.2%) patients were either active (40%) or former (41.2%) smokers, and the mean smoking history was 47.6 pack-years (SD: 25). A total of 12 patients (14.1%) had stage II COPD and 7 patients (8.2%) had stage III COPD, as shown in Table 1.

### 3.2. Perioperative

In 60 patients (70.6%), one lung segment was removed, followed by 15 (17.6%) and 10 (11.8%) who had 2 and 3 segments removed, respectively (Table 2). The mean duration of surgery was 126 (SD: 39) minutes (range 57 to 233 min).

Regarding lymph nodes harvested during surgery, the mean number of extracted lymph node stations and lymph nodes removed was 4.3 (SD: 1.8) and 8.9 (SD: 7.2), respectively.

The median duration of chest drainage was 1 day (1.-3. IQR 1–2), whereas the median length of hospital stay was 3 days (1.-3. IQR 3–5). Most patients were discharged home, and only two went to a rehabilitation clinic.

### 3.3. Postoperative Morbidity

A total of 13 patients (15.3%) experienced a postoperative complication that accounted for 30-day morbidity. The 30-day and in-hospital mortality rate was 1.2%, with 1 death (Table 3).

Postoperative complications included 5 cases of prolonged air leak (>5 days), 4 of which were reoperated 7, 8, 10, and 12 days after initial surgery, respectively. In one case, hemothorax was treated successfully with chest tube insertion. Moreover, two patients with pneumonia were treated conservatively with antibiotics.

In addition, three patients had radiologically significant pneumothorax after chest tube removal, and one of them required reinsertion of a chest tube under local anesthesia. One patient suffered severe nausea and vomiting postoperatively, and one developed a urinary tract infection.

The in-hospital mortality rate was 1.2%, with one death (Table 3). The deceased patient suffered acute bleeding from a preexisting duodenal ulcer and required emergency laparotomy on postoperative day eight. After a prolonged stay in the intensive care unit and repeat abdominal procedures, the patient died on postoperative day forty.

#### 3.3.1. Men and Women

There was a clinically significant difference in the rate of postoperative complications between men and women: 12.7% in men and 20% in women. However, this difference was not statistically significant (*p* = 0.52). Of note, complications appeared to be more severe in the male group, and all four patients who required reoperation for prolonged air leakage were men and active smokers.

There was no difference in mean age, duration of chest drainage, and length of hospital stay between the two sexes.

#### 3.3.2. Smoking History

Regarding smoking status and the clinical outcome, the proportion of active smokers was 43.6% in men versus 33.3% in women. The complication rate per se was not increased in the active smoker group, with 13.2% versus 16.7% (2 patients) in the never smoker group. Both patients in the group of never smokers who experienced a complication had a radiologically significant pneumothorax.

Moreover, there was a trend toward higher complication rates in patients with a smoking history of more than 40 pack-years and those with less than 40 pack-years (py). The complication rate in smokers with more than 40 py was 18% compared to 10.2% in the patient group with less than 40 py (*p* = 0.37)

### 3.4. Survival, Tumor-Related Deaths, and Recurrence

Lung adenocarcinoma was the most common histological type with 65 cases (76.5%), followed by squamous cell carcinoma with 14 cases (16.5%). In most cases, the tumors of 45 patients (54.2%) were in stage IA2 (according to the 8th edition of UICC), followed by 21 cases (25.3%) in stage IA1 and 17 cases (20.5%) in stage IA3.

One of the primary endpoints of this study was 3-year overall survival (OS), defined by the time interval between the date of surgery and either death or the last medical follow-up in our institution. Kaplan–Meier curves were analyzed by histological stage and substages.

At the end of the follow-up period, two patients were no longer included in the follow-up. Throughout the study period, 18 out of 83 (21.7%) patients died; 3 (16.7%) were stage IA1, 9 (50%) were stage IA2, and 6 (33.3%) were stage IA3.

**Figure 1:**
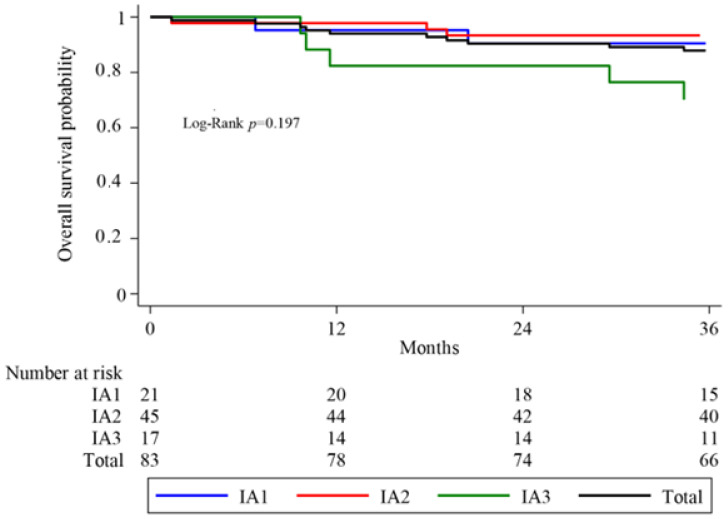
Kaplan–Meier analyses of overall survival (OS) in total sample and by group.

The 3-year cumulative OS rate was 87.9% for the entire cohort. It was 90.5% in the IA1 group, 93.3% in the IA2 group, and 70.1% in the IA3 group, respectively, (Figure 1).

The median overall survival of the entire cohort was 54 months (1.-3. IQR 43.5- 65.5). Between substages, the distribution was 57.1 months (1.-3. IQR 42–67), 58 months (1.-3. IQR 46–66), and 45.4 months (1.-3. IQR 32–57) for IA1, IA2, and IA3, respectively.

A log-rank test was performed to detect differences in survival between groups. No significant difference in overall survival was found between the three histological substages (*p* = 0.197) (Figure 1).

There were four tumor-related deaths in our cohort. Three patients died due to metastatic disease to the brain, while one patient developed pleural carcinomatosis.

As for the mortality rate in the subgroup of stage IA3 patients, six deaths were observed, two of which were tumor-related. One patient developed brain metastases, and the second had bone and pleural metastases. In the other four patients who died of non-tumor-related causes, the causes were as follows: Three patients died of cardiac failure due to the progression of preexisting coronary artery disease and valvular cardiopathy. One patient died due to lymphoma, diagnosed one year after lung resection.

Two cases of locoregional metastases were observed in our cohort, both in the same lobe of the initial segmentectomy. One case with suspected hilar recurrence is under active surveillance and the second patient, who had recurrence following posterior segmentectomy of the right upper lobe, underwent lobectomy eleven months after the initial surgery.

## 4. Discussion

Our study has several limitations. First, the retrospective nature of the study cannot exclude selection bias, and the patient selection was not thoroughly investigated in the current study. Furthermore, the sample size is relatively small with 85 patients, as we aimed to create a homogeneous population and have a follow-up period of at least three years.

In addition, the selection criteria between segmentectomy and lobectomy are not fully clear in all cases. Most cases involved intended segmentectomy; however, there were also many compromised segmentectomies in patients who would not have otherwise tolerated a lobectomy. This proportion of patients was higher in the T1c subgroup, including tumors larger than 2 cm, thus being, per definition, compromised segmentectomies.

Our monocentric study aimed to analyze the oncologic outcome of a homogeneous oncologic population of patients with pathological stage IA NSCLC after applying a standardized surgical technique uniformly. The main focus was on the oncologic outcome over a surveillance interval of at least three years after surgical therapy, and additionally on the clinical postoperative outcome. To our knowledge, no study has been performed in Europe to evaluate oncologic outcomes after uniportal segmentectomy in a homogeneous cohort in relation to surgical technique over this surveillance period. A recent study from Darras et al. [24] showed comparable local control rates after VATS segmentectomy and lobectomy for tumors smaller than 2 cm. However, the follow-up period was relatively short, and the study included uniportal and multiportal VATS segmentectomies. A more recent publication from the same centers, concerning only tumors from 2 to 3 cm, showed comparable results between segmentectomy and lobectomy [25]. Nevertheless, the follow-up period was short, and a significant proportion of patients were lost to the follow-up. As most survival studies evaluate 5-year overall survival as the primary endpoint, a comprehensive comparison with 3-year overall survival was difficult in our data set. Ijsseldijk et al. [10], in 2020, reported a 3-year overall survival of 82% after VATS lobectomy in a high-risk patient population with stage IA NSCLC, similar to the survival observed in our study (87.9%).

Regarding the 5-year overall survival (OS), survival rates of 78% to 100% after VATS lobectomy for stage I NSCLC have been reported in the existing literature [19,26,27]. We hypothesize that a higher comorbidity rate may also be a significant factor in the overall survival reported in our study. Tumor size and biological tumor characteristics are other important factors that have been shown to influence overall survival after limited resection in an older study [28]. When comparing 5-year overall survival, the outcome was significantly better in well-differentiated adenocarcinomas than in poorly differentiated adenocarcinomas. In our analysis, squamous cell carcinoma showed a trend toward a worse prognosis, but this did not reach statistical significance.

A recently published phase 3 multicenter randomized controlled trial by Saji et al. [14] found that segmentectomy outperformed lobectomy in terms of 5-year overall survival for peripheral tumors less than 2 cm in size (stage IA NSCLC, according to the 7th edition of UICC). However, locoregional recurrence occurred more frequently after segmentectomy compared with lobectomy. The results from our small cohort do not suggest higher recurrence rates following segmentectomy and are comparable to previously published data [29]. A systematic review and meta-analysis of 28 studies that compared lobectomy with segmentectomy for overall survival was consistent with the JCOG trial. Winckemans et al. [30] found no significant difference in OS between segmentectomy and lobectomy for Stage IA < 2 cm.

On the other hand, a single-institution retrospective study by Chan et al. in 2019 [31] showed similar 5-year overall survival in tumors 2–3 cm N0 after lobectomy and segmentectomy.

In the current study, an unexpectedly low (70%) 3-year overall survival was observed in subgroup IA3 (tumors 2–3 cm). The fact that most of these lung segmentectomies were compromised, as tumors larger than 2 cm are indicated for lobectomy if the patient is medically able to tolerate one, in combination with the small size of this subgroup, might be the answer to the low 3-year overall survival in stage IA3 patients. Nonetheless, two out of six deaths were tumor-related, meaning that the majority were non-tumor-related. This could further influence the results. It is thus impossible to rule out that the observed differences are due to factors other than the surgical technique.

Even if these results are somewhat inconclusive, these survival rates do not show a large discrepancy from the previously reported survival rates after segmentectomy for T1c. [32]. Yu et al. showed that the overall survival was significantly worse in the segmental resection group than those in the lobectomy group after comparing 400 segmentectomies with 9100 lobectomies.

VATS is associated with fewer postoperative complications, less postoperative pain, shorter hospital stays, and better quality of life than open surgical procedures [17,18,33]. In a large database study, Boffa et al. examined morbidity after VATS compared with open lobectomy for stage I lung cancer [33]. The perioperative complication rate was 30% for VATS and 36% for thoracotomy. The previously reported 30-day morbidity after uniportal VATS lobectomy [18] suggests that the perioperative complication rate is lower with the uniportal approach than with the multiport approach. A well-designed prospective randomized study assessing the perioperative complications, postoperative pain, and quality of life after uniportal compared to multiport VATS segmentectomy is needed to shed light on this issue.

Our findings are consistent with previously reported morbidity. The major contributors to 30-day morbidity in this study were prolonged air leak, reinsertion of chest tube, and pneumonia. Previous studies indicated the same main perioperative complications after VATS anatomical resection [7,19,34]. In a large, randomized trial evaluating the safety of segmentectomy versus lobectomy, Suzuki et al. [17] found no difference in perioperative complications between the different surgical approaches but observed a higher rate of air leakage in the segmentectomy group (6.5%). This rate correlates with our cohort (6%).

Regarding the number of ports, there are studies from North America and China [35,36] that included a large number of patients and showed similar perioperative complication rates with uniportal versus multiportal segmentectomy. Nonetheless, oncological outcomes were not analyzed. Zhou et al. [37] analyzed 2630 patients after uniportal and triportal segmentectomy and found similar overall survival and progression-free survival rates. The patient population was heterogeneous regarding surgical technique, and the cohort included a high number (>50%) of minimally invasive and in situ adenocarcinomas, which have a much more favorable oncological prognosis.

Furthermore, in agreement with the results of Bédat et al., we found no correlation between operative time and postoperative 30-day morbidity [34].

Another critical aspect of minimally invasive segmentectomies as a curative treatment for early-stage lung cancer is lymph node dissection. According to current knowledge, segmentectomy should be performed only in cases without lymph node involvement. More specifically, locoregional and hilar lymph nodes harvested during surgery should be sent for frozen section to minimize the postoperative upstaging rate. The number of lymph nodes removed during minimally invasive thoracoscopic lung resections is controversial and considered one of the main disadvantages of curative thoracoscopic lung resection in patients with lung cancer. A lower incidence of nodal upstaging (the presence of unsuspected nodal metastases in histopathological surgical specimens) has been reported as a potential disadvantage of segmentectomies in the treatment of T1 NSCLC [12,38]. When comparing thoracotomy and VATS lobectomy, nodal upstaging was significantly higher after thoracotomy (13.1% versus 8.1% for N1 and 11.5% versus 3.8% for N2) [38].

## 5. Conclusions

In conclusion, our initial experience with uniportal VATS segmentectomy for pathological stage IA NSCLC suggests a good short-term clinical outcome with low 30-day morbidity, making it a safe and feasible therapy in early-stage lung cancer. The 3-year overall survival is also promising and needs further investigation in the coming years, although survival was lower in the subgroup of stage IA3 patients than expected.

## Figures and Tables

**Table 1:** Patients’ characteristics.

	Total Sample
Age (years), Mean (SD)	66.4 (9.3)
FEV_1_ (L), Mean (SD)	2.3 (0.7)
COPD, N (%)	
No	58 (68.2)
Stage I	8 (9.3)
Stage II	12 (14.1)
Stage III	7 (8.2)
Smoking, N (%)	
Never smoker	16 (18.8)
Former smoker	35 (41.2)
Active Smoker	34 (40.0)
Pack-years, Mean (SD)	47.6 (25)

**Table 2:** Segments removed.

	Right			Left		
	N	%		N	%	
1	12	14.1		1	1.2	
2	3	3.5		2	2.4	
3	5	5.9		3	3.5	
5				1	1.2	
6	10	11.8		9	10.6	
8	3	3.5		4	4.7	
9				1	1.2	
10				4	4.7	
1–2	3	3.5		7	8.2	
1–3				10	11.8	
3–5				2	2.4	
8–9	1	1.2				
9–10	2	2.4		1	1.2	
7–10	1	1.2				
Total	40	47.1%		45	52.9%	

The mean tumor diameter was 1.6 cm (SD: 0.6).

**Table 3:** Postoperative Results.

OP-Duration, Mean (SD)	126.5 (38.8)
Histology, N (%)	
Adenocarcinoma	65 (76.5)
Squamous Cell Carcinoma	14 (16.5)
Other	6 (7.1)
Segments removed, N (%)	
1	60 (70.6)
2	15 (17.6)
3	10 (11.8)
Tumor diameter, Mean (SD)	1.58 (0.64)
Lymph Nodes harvested, Mean (SD)	8.94 (7.24)
Chest Drain Duration, Mean (SD)	2.04 (2.19)
30-day morbidity, N (%)	
No	72 (84.7)
Yes	13 (15.3)
In-hospital mortality, N (%)	
No	84 (98.8)
Yes	1 (1.2)

## Data Availability

The data presented in this study are available on reasonable request from the corresponding author. The data are not publicly available.
